# Results from the Population-Based Gutenberg Health Study Revealing Four Altered Autoantibodies in Retinal Vein Occlusion Patients

**DOI:** 10.1155/2020/8386160

**Published:** 2020-07-29

**Authors:** Katharina Bell, Vanessa M. Beutgen, Stefan Nickels, Katrin Lorenz, Yvonne Scheller, Hisham Elbaz, Tunde Peto, Katharina A. Ponto, Andreas Schulz, Philipp S. Wild, Thomas Münzel, Karl J. Lackner, Irene Schmidtmann, Manfred Beutel, Norbert Pfeiffer, Franz H. Grus, Alexander K. Schuster

**Affiliations:** ^1^Department of Ophthalmology, University Medical Center of the Johannes Gutenberg-University Mainz, Mainz, Germany; ^2^Department of Ophthalmology, Otto-Von-Guericke University, Magdeburg, Germany; ^3^Centre for Public Health, Queen's University Belfast, Belfast, UK; ^4^Center for Thrombosis and Hemostasis (CTH), University Medical Center of the Johannes Gutenberg-University Mainz, Mainz, Germany; ^5^Preventive Cardiology and Preventive Medicine/Center for Cardiology, University Medical Center of the Johannes Gutenberg-University Mainz, Mainz, Germany; ^6^German Center of Cardiovascular Research (DZHK), Partner Site Rhine-Main, Mainz, Germany; ^7^Center for Cardiology I, University Medical Center of the Johannes Gutenberg-University Mainz, Mainz, Germany; ^8^Department for Clinical Chemistry and Laboratory Medicine, University Medical Center of the Johannes Gutenberg-University Mainz, Mainz, Germany; ^9^Institute of Medical Biostatistics,Epidemiology and Informatics, University Medical Center of the Johannes Gutenberg-University Mainz, Mainz, Germany; ^10^Department of Psychosomatic Medicine and Psychotherapy, University Medical Center of the Johannes Gutenberg-University Mainz, Mainz, Germany

## Abstract

**Purpose:**

Retinal vein occlusion (RVO) is the second most common retinal vascular disease and a major cause of visual impairment. In this study, we aimed to observe whether RVO cases have different antibody profiles as a new potential risk factor and whether a conversion of retinal vein occlusion (RVO) to neovascular glaucoma (NVG), one of the major complications, is occurring within a 5-year timeframe.

**Methods:**

We performed a nested case-control study (1 : 4) within the Gutenberg Health Study (GHS), a population-based, prospective cohort study in the Rhine-Main Region of Germany including 15,010 participants. RVO subjects (*n* = 59) were identified by grading of fundus photographs. Optic nerves of RVO subjects and age- and sex-matched controls (*n* = 229) at baseline and their follow-up examination after 5 years were analyzed for glaucomatous alterations. Of all RVO subjects and controls, serum autoantibody profiles were measured using in-house manufactured antigen-antibody microarrays.

**Results:**

Of the 59 RVO patients, 3 patients (5%) showed glaucomatous optic disc alterations at baseline, whereas no new glaucoma case was detected at 5-year follow-up. Four of the autoantibodies measured (against dermcidin, neurotrophin-3, superoxide dismutase 1, and signal recognition particle 14 kDa protein) were significantly increased in the serum of RVO patients (*p* < 0.001). Multivariable conditional logistic regression analysis showed that 3 of these 4 antibodies were independent of cardiovascular risk factors.

**Conclusions:**

We found several autoantibodies associated with RVO, targeting proteins and structures possibly involved in RVO pathogenesis.

## 1. Introduction

Retinal vein occlusion (RVO) is the second most common retinal vascular disease and a major cause of visual impairment [1, 2]. The prevalence of RVO in the German population is 0.4%, and men are 1.7 times more frequently affected than women according to the population-based Gutenberg Health Study (GHS) [3]. Cardiovascular disease, certain coagulopathies, and ophthalmic parameters have been identified as risk factors for RVO [4]. Other factors such as alterations of cytokines, involvement of endothelin-1, and other inflammatory regulators are supposed to play a role in RVO, especially for the development of macular edema after RVO [5–7]. RVO can be differentiated by the position of occlusion, either as branch retinal vein occlusion (BRVO) or as central retinal vein occlusion (CRVO). Of all CRVO cases, approximately 20% are of the ischemic type [8] and potentially threatened to develop neovascular glaucoma (NVG).

Glaucoma is a group of conditions characterized by progressive optic nerve degeneration and loss of the visual field, ultimately resulting in blindness. Open-angle glaucoma has an estimated prevalence of 3.5% or 64 million affected people in 2013 worldwide and will increase to an estimated number of 116 million people affected by the year 2040 [9]. NVG is rare and accounts for around 3.9 % of all glaucoma cases, but it requires urgent, active management if sight is to be saved. NVG is characterized by iris and angle neovascularization, accompanied by aqueous humor outflow obstruction, resulting in sudden and severely elevated intraocular pressure (IOP). NVG is mainly caused by severe posterior segment ischemia, commonly due to proliferative diabetic retinopathy, CRVO, and BRVO, as well as ocular ischemic syndrome [10, 11], next to many other less common causes of NVG. Those with nonischemic CRVO are also at risk, as up to 30% can convert to ischemic CRVO in the following 3 years after the incidence of RVO [8] although the numbers vary here from 18.6% to 30% depending on the study.

NVG secondary to CRVO has been named the 90- or 100-day glaucoma, as it often occurs during this short time period after the initial RVO event. Nevertheless, it can occur as early as 2 weeks after the event, or even up to several years later [12]. Treatment of NVG due to CRVO is challenging, and especially the time point of treatment is essential in order to prevent complications of NVG and save sight. In brief, treatment of RVO is dependent on the occurrence and the size of retinal ischemia or on macular edema. Panretinal photocoagulation is frequently applied for retinal ischemia which can be associated with the occurrence of neovascularization on the iris or in the anterior chamber angle [13]. Anti-VEGF treatments are most commonly applied to treat RVO-associated macular edema [14, 15]; however, intravitreal steroids such as triamcinolone can also serve as a valuable treatment option for RVO-induced macular edema [16, 17] and other forms of edema [18]. Treatment of RVO has intensely been discussed in previous reviews [19, 20].

Some of our understanding on NVG after RVO comes from studies conducted in the USA, Asia, and Israel [21–23]. However, data on rates of conversion from RVO to NVG are scarce in European cohorts, as most studies had no follow-up data and therefore could only focus on RVO prevalence.

Using data from the GHS, a population-based, prospective cohort study was conducted in Germany with a total of 15,010 participants, and this study aims to analyze the proportion of glaucoma in general and furthermore possible conversion to NVG in subjects with RVO. Additionally, we aim to determine antibody profiles of those subjects. Our previous studies demonstrated that we can detect glaucoma by measuring serum autoantibodies (AABs) in a microarray approach with a sensitivity and specificity of over 93% and also show changes of AABs in longitudinal studies, such as after acute angle-closure glaucoma attack [24, 25]. By measuring the antibody profiles of RVO subjects, we therefore want to determine whether these provide any additional evidence towards other risk factors with additional focus on some glaucoma antibodies known from our previous studies.

## 2. Methods

### 2.1. Participants

The Gutenberg Health Study (GHS) is a population-based, prospective, observational, single-center cohort study in midwestern Germany that includes consecutive follow-ups every 5 years [26]. The baseline examination took place from 2007 to 2012. 15,010 participants aged 35–74 years underwent an ophthalmological examination and several cardiovascular and general examinations, as well as interviews and questionnaires. The study participants were randomly drawn and equally stratified for sex and each decade of age via the local residents' registration offices. The study protocol and study documents were approved by the Local Ethics Committee of the Medical Chamber of Rhineland-Palatinate, Germany (reference no. 837.020.07; original vote: 22.3.2007; latest update: 20.10.2015). According to the tenets of the Declaration of Helsinki, written informed consent was obtained from all participants prior to their entry into the study. Based on the GHS population, we performed a nested case-control study to analyze serum autoantibody profiles in RVO subjects and population-based controls.

### 2.2. Measurements

#### 2.2.1. Ophthalmic Data

All participants underwent a standardized protocol with a general cardiovascular and ophthalmic examination including objective refraction and corrected visual acuity (Humphrey^®^ Automated Refractor/Keratometer (HARK) 599™; Carl Zeiss AG, Jena, Germany), noncontact tonometry (Nidek NT-2000™, Nidek Co., Japan), and nonmydriatic fundus images (Visucam^®PRO NM^; Carl Zeiss AG, Jena, Germany). For a detailed description of the ophthalmic study design, see Höhn et al. [27].

Fundus images were taken with a nonmydriatic fundus camera in a darkened room and with the pupil's natural width. Grading of fundus images of the GHS for RVO was carried out by a trained grader (HE) and supervised by an experienced clinician grader (TP), as described previously [3]. In brief, using 30° and 45° photographs centered on the optic nerve and a 30° photograph centered on the macula, signs of RVO such as retinal thickening, hemorrhages, abnormal retinal vessel caliber, or the appearance of arteriovenous collaterals were determined in a standardized grading procedure. Fundus images of sufficient quality of at least one eye to allow for classification were available in 12,954 (86.3%) of GHS participants. For optic nerve head analysis, in a masked fashion, two board-certified ophthalmologists independently analyzed the optic nerve head images of RVO cases, as well as the corresponding controls at baseline and the 5-year follow-up examination on the presence of glaucomatous alterations. Two color and brightness standardized computer screens were used for image analysis in standardized mesopic light conditions. In case of different findings, a third board-certified ophthalmologist reviewed the cases.

#### 2.2.2. Autoantibody Data

Full blood samples, drawn at the baseline visit, were allowed to clot for 30 minutes and were centrifuged for 10 minutes at 4°C and 1000 × g. The supernatant was collected in Eppendorf tubes, and serum samples were stored at −80°C. After thawing, the individual serum samples were diluted at the ratio 1 : 250 with phosphate-buffered saline (PBS). 100 *µ*l of the diluted sample was used per included subject and microarray measurement. Within this study, antigen-antibody microarrays were performed, as outlined below.

#### 2.2.3. Microarray Fabrication

The required antigen-antibody microarray slides were manufactured in-house using a noncontact piezo-dispenser (SciFLEXARRAYER S3, SCIENION, Berlin, Germany) and processed as described before [25]. To measure the antibodies in the samples, antigens need to be spotted onto the slides and then incubated with the antibody-containing sample. For this, selected antigens (50 antigens were chosen for this study) were purchased as purified, recombinant human proteins. Proteins were spotted in triplicates onto nitrocellulose-coated microarray slides (AVID ONCYTE, 16 Pad NC slides, Grace Bio-Labs, Bend, Oregon, USA). Next, to the samples, human IgG (Sigma) and secondary antibody conjugated to Alexa Fluor 647 were included on each array as the control. Spotting was performed in a humidity chamber with humidity set to 60%. Slides were kept on the spotter platform to dry overnight prior to incubation to facilitate protein immobilization on the nitrocellulose.

#### 2.2.4. Microarray Incubation and Image Acquisition

All slides were incubated using 16-pad incubation chambers (ProPlate Multiwell chambers, Grace Bio-Labs, Bend, USA). For best and reproducible results, all incubation steps were performed at 4°C on an orbital shaker. At first, arrays were incubated with the blocking buffer (Super G, Grace Bio-Labs, Bend, Oregon, USA) for 1 hour and washed 3 times with phosphate-buffered saline containing 0.5% Tween 20 (PBST) before incubating the arrays with 100 *µ*L of the diluted serum samples overnight. One subarray on each slide was randomly selected as the negative control and was incubated with PBS. Subsequently, the slides were washed again 3 times with PBST which was followed by the incubation with a secondary anti-human antibody conjugated with Alexa Fluor 647 (Alexa Fluor® 647 AffiniPure Goat Anti-Human IgG, Fc*γ* fragment specific, 109-605-008, Jackson Immunoresearch; 1 : 500 in PBS) for 1 h at room temperature (RT), allowing visualization of the bound antibodies. The nonbound secondary antibody was then removed by washing the slides twice each with PBST and ultrapure water. Microarray slides were dried for 2 minutes in a vacuum concentrator (SpeedVac, Thermo Scientific, Waltham, Massachusetts, USA). For analysis, the slides were scanned with a high-resolution confocal laser scanner (428 Array Scanner, Affymetrix, Santa Clara, California, USA), and images were saved as 16-bit TIFF. All spot intensities were quantified with the image analysis software Imagene (Imagene 5.5, BioDiscovery Inc., Los Angeles, California, USA). All slides were checked, and spots with poor quality were manually flagged and removed from further analysis.

#### 2.2.5. Microarray Data Preprocessing

To eliminate the background noise, local background was subtracted from the median spot intensities. In addition, to correct for the unspecific binding of the secondary antibody, the signal of the negative control included on each slide was subtracted from each spot. Negative background subtracted intensities were excluded from analysis. Each antigen was spotted in triplicate, and the replicated spot intensities were averaged, yielding one mean fluorescence intensity. To correct for technical variability, the signal intensities were normalized to the IgG (Immunoglobulin G) control spots on each subarray by median centering. To do so, the IgG median signal intensities were divided by the overall IgG signal median, yielding a normalization factor. All signal intensities on one subarray were multiplied with their corresponding normalization factor. The resulting normalized fluorescence intensities (NFI) were forwarded to statistical analysis. Variables with more than 30% missing data were excluded. Two batches of microarray analysis were carried out, as 23% of the blood samples were delivered at a later time point.

### 2.3. General and Cardiovascular Data

Smoking was dichotomized into nonsmokers (never smokers and ex-smokers) and smokers (occasional and regular smokers). Arterial hypertension was diagnosed if antihypertensive medication was taken, in case of the diagnosis of hypertension by a physician, in cases with a mean systolic blood pressure of ≥140 mmHg in the 2nd and 3rd standardized measurement (Omron HEM 705-CP II, OMRON, Mannheim, Germany) after 8 and 11 minutes of rest, respectively, or a mean diastolic blood pressure of ≥90 mmHg in the 2nd and 3rd standardized measurement after 8 and 11 minutes of rest, respectively. Diabetes was diagnosed when either HbA1c ≥6.5%, an intake of antidiabetic medication, or diagnosis of diabetes by a physician was reported. Obesity was defined as a body mass index (BMI) ≥30 kg/m^2^. Dyslipidemia was defined as an LDL/HDL ratio of ≥3.5 or diagnosis by a physician or intake of lipid-modifying medication.

### 2.4. Statistical Analyses

For each RVO case, we selected 4 age- and sex-matched controls without RVO from the same GHS population and batch.

We used the Mann–Whitney *U* test to explore the association of AAB levels with RVO status. We used a Bonferroni-corrected *p* value threshold of <0.001 (*n* = 50 AABs) to select potentially associated AABs. For these AAB candidates, we inspected the distribution using box plots. Descriptive statistics included means and standard deviations, or medians and interquartile range. We used multivariable conditional logistic regression analysis to explore the association of RVO and AAB levels. For this, we log10-transformed AAB levels. We calculated models adjusted for batch (model *#*1) and model *#*2 additionally adjusted for potential confounders (age, sex, obesity, arterial hypertension, diabetes, smoking, dyslipidemia, family history of myocardial infarction, or stroke as categorical traits), and model *#*3 adjusted for potential confounders (defined as continuous variables: age, sex, BMI, mean arterial blood pressure, HbA1c, HDL, LDL, triglycerides, smoking, family history of myocardial infarction, or stroke).

Analyses were performed using *R* version 3.5.2 (a language and environment for statistical computing *R* Foundation for Statistical Computing, Vienna, Austria, URL http://www.R-project.org).

## 3. Results

### 3.1. Study Population

Our study sample consisted of 59 RVO cases (CRVO and BRVO) and 229 controls matched by age and sex. In the RVO group, three subjects had glaucoma at baseline examination. AAB measurements were not available in one of the 59 RVO cases within the GHS. For 55 of the remaining 58 RVO cases, AAB measurements in the 4 matched controls were successful; for 3 RVO cases, AAB measurements in only 3 of the 4 matched controls were successful.  Table 1 shows the main characteristics of the included population. There were no apparent differences in ophthalmic measures such as intraocular pressure (IOP) and spherical equivalent. The RVO study group descriptively had a higher frequency of cardiovascular diseases and risk factors, see Table 1.

Optic nerve head analysis was performed on pictures taken at baseline and the 5-year follow-up with the aim to detect newly occurred glaucoma in the RVO patient population. The optic nerve head analysis revealed that there were no cases of new-onset glaucoma in both the RVO and the control groups within the 5-year observation time.

### 3.2. Autoantibody Measurements

All of the analyzed AABs could be detected in the samples. Due to technical reasons, AAB measurements were performed in 2 batches. The first batch included 54 cases and 168 controls, and the second batch had 4 cases and 61 controls.

**Figure 1:**
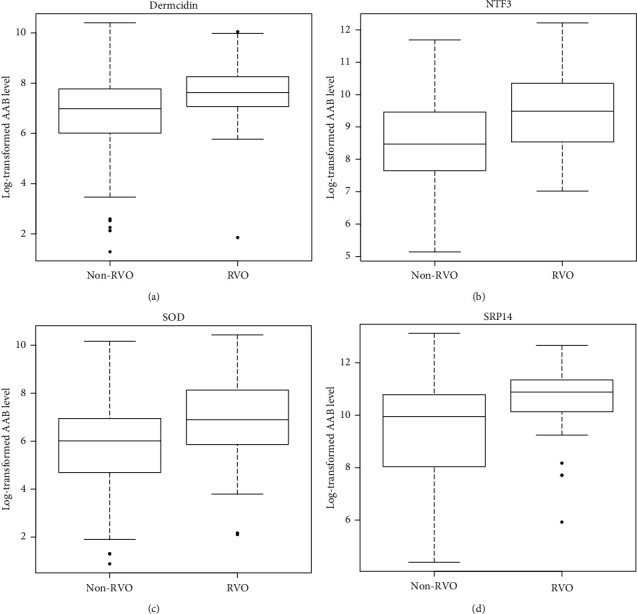
Distribution of significantly altered autoantibody levels in the analysis sample of the German population-based Gutenberg Health Study (2007–2012) stratified on retinal vein occlusion status. The boxplots of the 4 AABs that show significantly different levels in the RVO population in comparison with the control population are presented (*p* value <0.001). Log-transformed normalized fluorescence intensity of these autoantibodies targeted against (a) dermcidin, (b) neurotrophin-3, (c) superoxide dismutase 1, and (d) signal recognition particle 14 kDa protein is displayed.

When comparing RVO with non-RVO measurements, we found several AABs (against dermcidin (DCD), neurotrophin-3 (NFT3), superoxide dismutase 1 (SOD1), and signal recognition particle 14 kDa protein (SRP14), see Table 2 and Figure 1) that had a significantly different abundancy (*p* < 0.001). AABs against all these antigens were found with an increased abundance in the serum of RVO patients. Multivariable conditional logistic regression analysis revealed that differences in NFT3, SOD1, and SRP14 remained significant when adjusting for cardiovascular risk factors (Table 3), while there was no association for DCD in adjusted analysis. Similar results were observed in the sensitivity analysis including only measures of batch 1. As none of the RVO population showed conversion to NVG, identifying people at risk by AAB analysis was not possible.

## 4. Discussion

This study includes 59 subjects presenting with RVO in the baseline visit of the GHS, which involved 15,010 participants with an age range of 35–74 years. Follow-up visits are performed every 5 years, allowing the unique possibility to collect longitudinal data. Our interest in this study was to determine the proportion of RVO subjects showing conversion to NVG from baseline to the consecutive follow-up visit and furthermore analyze AAB profiles in the serum of these subjects to determine possible pathogenically relevant targets.

### 4.1. No Conversion of RVO to NVG

At baseline, 3 subjects had glaucoma in the RVO group, and no new glaucoma case was detected after the 5-year follow-up period in this study sample. We therefore could not detect any RVO subject converting from RVO to NVG within this time period. Retinal ischemia is the main risk factor for developing NVG after RVO, whereas especially large ischemic retinal capillary nonperfusion areas (>5.5–10 disc areas) increase the risk of developing NVG [28]. However, retinal ischemia was not assessed in the context of this study, due to lack of fluorescein angiography or retinal widefield imaging within the study setting. In addition, our study population presented with CRVO and BRVO of unknown duration at baseline. Conversion rates to NVG after RVO differ within the literature. The SCORE study, a prospective randomized, multicenter clinical trial, shows an 8.5% incidence for NVG and neovascularization of the iris (NVI) after CRVO 36 months after the insult [28], independent of the treatment group. The CVOS (Central Vein Occlusion Study), a prospective randomized clinical trial, shows higher incidence rates of 16% conversion to NVI, and 8.5% of these patients develop NVG despite therapy with photocoagulation within the 3-year follow-up time [12]. Should NVG have occurred in one of the included RVO subjects, the time period of 5-year follow-up should be sufficient to detect conversion. A large retrospective study documented the mean time until NVG development after RVO of 421 days after the insult and 212 days after the last anti-VEGF injection with a cumulative probability for NVG of 13% after RVO [29]. Another retrospective study analyzing the effect of anti-VEGF on the occurrence of NVG after RVO showed an overall NVG conversion rate of 6.8% after a mean time of 19.7 months. The authors concluded that anti-VEGF does not prevent NVG but could delay the onset [30]. Although there is a small possibility of patients developing NVG at a later time point, we conclude that none of the subjects included in our cohort developed NVG so far. Due to limitations of the study design we additionally cannot rule out that we observed the initial timeframe after RVO onset, the timeframe with the highest risk of NVG development.

### 4.2. Autoantibodies Associated with RVO

So far, antiphospholipid autoantibodies and their subgroup, anti-cardiolipin autoantibodies, have been associated with RVO, which was summarized nicely in a recently performed meta-analysis [31]. The analysis performed within this study revealed additional AABs in the serum of the RVO population. The AABs found to be differently abundant in the RVO population include proteins involved in the oxidative stress response pathway as well as neuroprotective pathways.

One of the AABs is targeted against superoxide dismutase 1 (SOD1). SOD1 is an enzyme catalyzing the elimination or deactivation of the superoxide anion (O_2_^•−^) and which therefore is involved in preventing reactive oxygen species [32]. It is one of the antioxidant defense systems and plays an important role in vascular endothelial function [33]. Animal models show that decreased levels of SOD1 predispose for thrombosis [34]. Increased oxidative stress response markers and decreased levels of antioxidant enzymes, including SOD1, were found in the serum of RVO patients [35]. More specifically, erythrocytes of RVO patients show increased oxidative stress levels [36], probably contributing to reduced erythrocyte deformability in the retina. Platelet aggregation is relevant for blood viscosity and therefore is part of the Virchow triad which describes the 3 factors leading to venous thrombosis, these being abnormal endothelium (“abnormal vessel wall”), abnormal hemorheology (“abnormal blood flow”), and abnormalities in platelets and coagulation and fibrinolysis pathways (referring to Virchow's described “abnormal blood constitutes”) [37, 38]. Less is known about SRP14, which also was targeted by one of the significantly differently abundant AABs in RVO. It belongs to the cytoplasmic ribonucleoprotein complex called the signal recognition particle (SRP) and is involved in the recognition of secretory signals derived from the ribosome [39]. Proteomic analysis reveals the existence of SRP14 in human retinal endothelial cells [40]. One AAB found with a significantly altered abundance in RVO subjects is targeted against neurotrophin-3 (NFT3). Neurotrophin-3 belongs to the neurotrophin family of growth factors, which also includes NGF (nerve growth factor), BDNF, and neurotrophin-4/5. Neurotrophin-3 is relevant for neuronal and retinal development, including retinal ganglion cells and amacrine cells [41–43], and can be found in several cells throughout the retina [44]. NFT3 plays a role in neuronal survival after ischemic damage; however, the literature is controversial as to whether it is solely beneficial or rather detrimental. However, studies show that decreased NFT3 levels result in smaller infarct lesions in cerebral ischemia and increased NFT3 levels promote enhanced neuronal cell death [45], and other studies support NFT3 as a neuroprotective factor after cerebral ischemia/reperfusion [46].

The AABs were detected as significantly altered in our RVO population target proteins or pathways with biological feasibility in the pathogenesis of RVO, such as platelet aggregation or oxidative stress pathways. However, conclusions concerning causality of the AABs for the development of RVO or NVG cannot be drawn.

### 4.3. Strengths and Limitations

We present data from a standardized, population-based study design including approximately 15,010 participants at the baseline visit and identify RVO subjects from the general population. From this large study population, we drew age- and sex-matched population-based controls. In accordance with the literature, demonstrating systemic risk factors to be relevant for RVO [3, 4, 47], the distribution of cardiovascular risk factors such as dyslipoproteinaemia, arterial hypertension, or obesity was increased in our RVO study population in comparison with the non-RVO age- and sex-matched controls. The multivariable regression analysis showed that the detected AABs were independent of the presented cardiovascular risk factors. Additionally, categorization of the type of glaucoma in subjects at baseline was unknown, as well as the time point of the RVO event.

## 5. Conclusions

None of the prevalent RVO cases of this population-based study had converted to NVG within the 5-year follow-up period. However, we found several AABs (against neurotrophin-3, superoxide dismutase 1, and SRP14) associated with RVO, which target proteins and structures possibly involved in RVO pathogenesis.

## Figures and Tables

**Table 1:** Characteristics of the study sample of the German population-based Gutenberg Health Study (GHS), 2007–2012.

	No RVO (*n* = 229)	RVO (*n* = 58)
Age (years)	62.33 ± 9.49	62.36 ± 9.51
Female sex	82 (35.8%)	21 (36.2%)
Spherical equivalent OD (D)	−0.02 ± 2.26	−0.10 ± 2.23^*#*^
Spherical equivalent OS (D)	−0.05 ± 2.18	−0.10 ± 2.52^+^
Intraocular pressure OD (mm Hg)	13.69 ± 2.71	14.26 ± 2.98^*#*^
Intraocular pressure OS (mm Hg)	13.87 ± 2.69	14.86 ± 2.62^+^
Visual acuity OD (logMAR) (median; interquartile range)	0.10 [0.00, 0.20]	0.20 [0.00, 0.50]^*#*^
Visual acuity OS (logMAR) (median; interquartile range)	0.00 [0.00, 0.20]	0.10 [0.00, 0.20]^+^
Arterial hypertension (yes)	153 (66.8%)	46 (79.3%)
Diabetes (yes)	34 (14.8%)	7 (12.1%)
Dyslipidemia (yes)	96 (41.9%)	30 (51.7%)
Obesity (yes)	54 (23.6%)	19 (32.8%)
Smoking (yes)	28 (12.2%)	7 (12.1%)
Family history of myocardial infarction or stroke	49 (21.4%)	15 (25.9%)
BMI (kg/m^2^)	27.41 ± 4.15	28.62 ± 4.64
Mean arterial pressure (mm Hg)	101.02 ± 11.36	105.28 ± 16.61
HbA1c (%)	5.69 ± 0.70	5.79 ± 1.13
Triglycerides (mg/dl) (median; interquartile range)	111.00 [85.00, 150.44]	115.00 [87.75, 154.11]
HDL (mg/dl)	54.78 ± 15.58	56.99 ± 15.13
LDL (md/dl)	143.23 ± 34.76	142.18 ± 42.58

For categorical variables, absolute and relative frequencies are given, and for approximately normally distributed continuous variables, mean and standard deviation (mean ± SD), or median and interquartile range are given. OD = right eye, OS = left eye, ^*#*^eyes with RVO, and ^+^fellow eyes of subjects with RVO in the other eye.

**Table 2:** Measurement of autoantibody abundancies in the study sample of the German population-based Gutenberg Health Study (2007–2012).

Autoantibody	Missing values (%)	No RVO	RVO	*p* value
ACO2	6.3	2074 [728, 4173]	3160 [1677, 5522]	0.010
ACTA1	18.1	3036 [1473, 6445]	3178 [1903, 5223]	0.678
ALB	0.3	11092 [5271, 26362]	17087 [9570, 30900]	0.032
APOA1	23.7	16366 [8269, 31017]	17724 [12739, 33491]	0.158
CRYB	0.7	10868 [5054, 20103]	11281 [7384, 18234]	0.519
BDNF	1.0	8822 [3083, 20522]	12841 [5202, 19563]	0.231
CA2	23.0	38521 [18099, 66345]	51944 [29945, 91025]	0.011
CALR	10.1	2685 [1065, 8259]	4043 [1603, 11782]	0.024
CKB	24.0	132 [51, 385]	165 [70, 335]	0.844
CLUS	0.7	3754 [1896, 7731]	3829 [2254, 6237]	0.832
**DCD**	**27.5**	**1132 [451, 2406]**	**2063 [1190, 3707]**	**<0.001**
DPYSL2	1.7	1519 [661, 4406]	2759 [1460, 6229]	0.003
EIF4A1	13.2	3019 [1174, 7473]	4587 [1741, 8618]	0.086
ENO2	0.7	7902 [3529, 15917]	7836 [4756, 15253]	0.590
GAPDH	0.7	3744 [1915, 7447]	4692 [3010, 7110]	0.033
GFAP	0.3	3285.50 [1555.65, 7164.40]	5693.41 [2579.22, 8549.41]	0.009
GLUL	5.6	703.88 [285.72, 1616.39]	1547.99 [598.85, 2849.82]	0.002
GNB1	30.0	54.50 [17.62, 125.47]	26.88 [8.84, 90.49]	0.134
GPD2	18.1	3081.16 [1549.40, 7339.39]	3743.75 [2143.56, 7727.56]	0.216
groEL2	2.4	27782.73 [13044.47, 54270.02]	47373.91 [26874.14, 82068.28]	0.001
HARS	1.4	4597.49 [2318.39, 7798.38]	6565.70 [3958.94, 9588.00]	0.019
HSPB1	2.8	4617.03 [1732.16, 8855.58]	5408.40 [2816.20, 9088.60]	0.160
HSPD1	0.3	3299.42 [1401.20, 8817.68]	4461.00 [2463.66, 10410.47]	0.044
IGLL	25.4	811.69 [330.99, 1564.34]	1283.75 [714.98, 2346.66]	0.006
LYZ	16.0	555.54 [186.65, 7302.15]	263.15 [120.03, 704.77]	0.002
MAPK3	0.7	9830.88 [2458.02, 23857.96]	15368.74 [8359.28, 37116.40]	0.001
MUC5B	24.7	1066.18 [544.56, 1997.16]	1251.97 [640.10, 1880.22]	0.362
**NTF3**	**2.8**	**4853.19 [2039.01, 12903.88]**	**13178.46 [5119.04, 31061.95]**	**<0.001**
NTF4	6.3	883.80 [443.35, 1608.61]	1576.92 [788.47, 2718.02]	0.001
OGFR	3.1	1402.66 [696.57, 3144.92]	2163.76 [1257.49, 4107.68]	0.005
PDIA3	18.1	17431.70 [7605.95, 34366.26]	18777.89 [10221.49, 36333.68]	0.539
PKC	9.8	155.38 [70.51, 373.37]	188.74 [87.99, 438.85]	0.265
PPIA	0.7	4817.81 [2002.12, 10294.61]	5839.27 [2960.55, 10091.30]	0.155
PRKCSH	3.1	4898.71 [2353.30, 10513.01]	6255.10 [3161.41, 10863.94]	0.123
rhEPCR	0.7	44095.74 [25652.18, 85043.96]	64727.67 [32507.14, 105013.72]	0.052
SCFD1	1.7	4275.51 [2084.45, 11175.74]	6504.28 [3093.82, 17793.15]	0.032
SERPINA	2.8	1371.35 [692.52, 3150.89]	2362.25 [1633.70, 4431.63]	0.001
SNCA	3.5	1347.53 [647.41, 3002.53]	2669.78 [1139.49, 3811.30]	0.001
SNCG	2.4	29207.40 [9905.51, 61074.74]	35001.63 [20500.83, 63952.74]	0.098
**SOD1**	**23.0**	**407.98 [110.07, 1046.57]**	**988.49 [353.56, 3169.13]**	**<0.001**
SPTA1	0.3	2942.47 [1635.98, 5839.15]	4066.71 [2760.73, 6634.58]	0.035
**SRP14**	**3.8**	**21131.03 [3267.23, 49260.49]**	**53043.77 [25105.93, 84696.86]**	**<0.001**
TF	13.2	24740.36 [11311.13, 54334.60]	35316.44 [21494.71, 53916.44]	0.044
TG	0.3	48864.84 [28526.26, 87754.27]	68760.55 [40027.30, 112662.03]	0.013
TNNI3	2.1	2227.38 [1014.05, 4739.08]	2584.20 [1507.94, 4812.98]	0.178
TOP1	4.9	8365.60 [4257.88, 18528.31]	9320.98 [5320.86, 23541.94]	0.434
UCHL1	10.8	3014.20 [1257.18, 8253.46]	7115.37 [2077.31, 14879.76]	0.009
USP10	27.9	400.38 [91.67, 1374.01]	190.32 [68.20, 402.91]	0.027
VEGF	8.4	798.44 [319.16, 1490.63]	846.76 [536.98, 1588.95]	0.155
YWHAE	20.2	11895.96 [5219.60, 24643.01]	16298.38 [10034.07, 33682.41]	0.018

Median normalized fluorescence intensity (NFI) with interquartile range is shown for non-RVO and RVO samples.

**Table 3:** Association of autoantibody levels with retinal vein occlusion in the German population-based Gutenberg Health Study (GHS), 2007–2012.

Autoantibody	Model 1		Model 2		Model 3	
Log10-transformed autoantibody level (RVO: yes vs. no)	OR	*p*	OR	*p*	OR	*p*
Dermcidin	1.90 (0.89–4.09)	0.10	1.75 (0.79–3.88)	0.17	1.67 (0.75–3.72)	0.21
NFT3	2.44 (1.35–4.41)	0.003	2.64 (1.40–4.98)	0.003	2.49 (1.33–4.69)	0.005
SOD1	2.11 (1.21–3.68)	0.008	2.16 (1.21–3.85)	0.009	1.95 (1.08–3.51)	0.027
SRP14	2.65 (1.27–5.53)	0.009	2.70 (1.23–5.91)	0.013	2.79 (1.26–6.18)	0.011

Results are from conditional logistic regression analysis. Model 1 is adjusted for batch, model 2 is adjusted for age, sex, and cardiovascular risk factors (obesity, arterial hypertension, diabetes mellitus, smoking, dyslipidemia, family history of myocardial infarction or stroke, and batch), and model 3 is adjusted for age, sex, and cardiovascular risk factors (BMI, mean arterial blood pressure, HbA1c, HDL, LDL, triglycerides, smoking, family history of myocardial infarction or stroke, and batch). OR is the per log10-transformed autoantibody level.

## Data Availability

The written informed consent of GHS study participants does not approve public access to the data. This concept was requested by the local data protection officer and the ethics committee (Local Ethics Committee of the Medical Chamber of Rhineland-Palatinate, Germany). Access to data at the local database in accordance with the ethics vote is offered upon request at any time. Interested researchers can make their requests to the Principal Investigators of the Gutenberg Health Study (e-mail: info@ghsmainz.de).

## References

[1] Ehlers J. P., Fekrat S. (2011). Retinal vein occlusion: beyond the acute event. *Survey of Ophthalmology*.

[2] Suñer I. J., Margolis J., Ruiz K., Tran I., Lee P. (2014). Direct medical costs and resource use for treating central and branch retinal vein occlusion in commercially insured working-age and medicare populations. *Retina*.

[3] Ponto K. A., Elbaz H., Peto T. (2015). Prevalence and risk factors of retinal vein occlusion: the Gutenberg health study. *Journal of Thrombosis and Haemostasis*.

[4] Kolar P. (2014). Risk factors for central and branch retinal vein occlusion: a meta-analysis of published clinical data. *Journal of Ophthalmology*.

[5] Ascaso F. J., Huerva V., Grzybowski A. (2014). The role of inflammation in the pathogenesis of macular edema secondary to retinal vascular diseases. *Mediators of Inflammation*.

[6] Kida T. (2017). Mystery of retinal vein occlusion: vasoactivity of the vein and possible involvement of endothelin-1. *BioMed Research International*.

[7] Noma H., Yasuda K., Mimura T., Ofusa A., Shimura M. (2020). Relationship between retinal blood flow and cytokines in central retinal vein occlusion. *BMC Ophthalmology*.

[8] Hayreh S. S., Zimmerman M. B., Podhajsky P. (1994). Incidence of various types of retinal vein occlusion and their recurrence and demographic characteristics. *American Journal of Ophthalmology*.

[9] Kapetanakis V. V., Chan M. P. Y., Foster P. J., Cook D. G., Owen C. G., Rudnicka A. R. (2016). Global variations and time trends in the prevalence of primary open angle glaucoma (POAG): a systematic review and meta-analysis. *British Journal of Ophthalmology*.

[10] Shazly T. A., Latina M. A. (2009). Neovascular glaucoma: etiology, diagnosis and prognosis. *Seminars in Ophthalmology*.

[11] Rodrigues G. B., Abe R. Y., Zangalli C. (2016). Neovascular glaucoma: a review. *International Journal of Retina and Vitreous*.

[12] (1997). “Natural history and clinical management of central retinal vein occlusion. *Archives of Ophthalmology*.

[13] Central Vein Occlusion Study of Photocoagulation Therapy (1993). Baseline findings. *The Online Journal of Current Clinical Trials*.

[14] Campochiaro P. A., Brown D. M., Awh C. C. (2011). Sustained benefits from ranibizumab for macular edema following central retinal vein occlusion: twelve-month outcomes of a phase III study. *Ophthalmology*.

[15] Brown D. M., Heier J. S., Clark W. L. (2013). Intravitreal aflibercept injection for macular edema secondary to central retinal vein occlusion: 1-year results from the phase 3 COPERNICUS study. *American Journal of Ophthalmology*.

[16] Ip M. S., Scott I. U., VanVeldhuisen P. C. (2009). A randomized trial comparing the efficacy and safety of intravitreal triamcinolone with observation to treat vision loss associated with macular edema secondary to central retinal vein occlusion: the standard care vs corticosteroid for retinal vein occlusion (SCORE) study report 5. *Archives of Ophthalmology*.

[17] Haller J. A., Bandello F., Belfort R. (2010). Randomized, sham-controlled trial of dexamethasone intravitreal implant in patients with macular edema due to retinal vein occlusion. *Ophthalmology*.

[18] Scorolli L. (2007). Treatment of cystoid macular edema in retinitis pigmentosa with intravitreal triamcinolone. *Archives of Ophthalmology*.

[19] Abadia B., Calvo P., Ferreras A., Bartol F., Verdes G., Pablo L. (2016). Clinical applications of dexamethasone for aged eyes. *Drugs & Aging*.

[20] Ashraf M., Souka A. A. R., Singh R. P. (2016). Central retinal vein occlusion: modifying current treatment protocols. *Eye*.

[21] Rothman A. L., Thomas A. S., Khan K., Fekrat S. (2019). Central retinal vein occlusion in young individuals. *Retina*.

[22] Jonas J. B., Nangia V., Khare A., Sinha A., Lambat S. (2013). Prevalence and associations of retinal vein occlusions. *Retina*.

[23] Thapa R., Bajimaya S., Paudyal G. (2017). Prevalence, pattern and risk factors of retinal vein occlusion in an elderly population in Nepal: the Bhaktapur retina study. *BMC Ophthalmology*.

[24] Lorenz K., Beck S., Keilani M. M., Wasielica-Poslednik J., Pfeiffer N., Grus F. H. (2017). Course of serum autoantibodies in patients after acute angle-closure glaucoma attack. *Clinical & Experimental Ophthalmology*.

[25] Beutgen V. M., Perumal N., Pfeiffer N., Grus F. H. (2019). Autoantibody biomarker discovery in primary open angle glaucoma using serological proteome analysis (SERPA). *Frontiers in Immunology*.

[26] Wild P. S., Zeller T., Beutel M. (2012). The Gutenberg health study. *Bundesgesundheitsblatt Gesundheitsforschung Gesundheitsschutz*.

[27] Hohn R., Kottler U., Peto T. (2015). The ophthalmic branch of the Gutenberg Health Study: study design, cohort profile and self-reported diseases. *PLoS One*.

[28] Chan C. K., Ip M. S., Vanveldhuisen P. C. (2011). SCORE Study report #11: incidences of neovascular events in eyes with retinal vein occlusion. *Ophthalmology*.

[29] Rong A. J., Swaminathan S. S., Vanner E. A., Parrish R. K. (2019). Predictors of neovascular glaucoma in central retinal vein occlusion. *American Journal of Ophthalmology*.

[30] Ryu C. L., Elfersy A., Desai U. (2014). The effect of antivascular endothelial growth factor therapy on the development of neovascular glaucoma after central retinal vein occlusion: a retrospective analysis. *Journal of Ophthalmology*.

[31] Zhu W., Wu Y., Xu M. (2014). Antiphospholipid antibody and risk of retinal vein occlusion: a systematic review and meta-analysis. *PLoS One*.

[32] Miller A.-F. (2012). Superoxide dismutases: ancient enzymes and new insights. *FEBS Letters*.

[33] Fukai T., Ushio-Fukai M. (2011). Superoxide dismutases: role in redox signaling, vascular function, and diseases. *Antioxidants & Redox Signaling*.

[34] Dayal S., Gu S. X., Hutchins R. D. (2015). Deficiency of superoxide dismutase impairs protein C activation and enhances susceptibility to experimental thrombosis. *Arteriosclerosis, Thrombosis, and Vascular Biology*.

[35] Chen K. H., Hsiang E. L., Hsu M. Y. (2019). Elevation of serum oxidative stress in patients with retina vein occlusions. *Acta Ophthalmologica*.

[36] Becatti M., Marcucci R., Gori A. M. (2016). Erythrocyte oxidative stress is associated with cell deformability in patients with retinal vein occlusion. *Journal of Thrombosis and Haemostasis*.

[37] Chung I., Lip G. Y. (2003). Virchow’s triad revisited: blood constituents. *Pathophysiol Haemost Thromb*.

[38] Virchow R. (1856). *Thrombose und Embolie. Gefässentzündung und septische Infektion*.

[39] Gorodkin J. (2001). SRPDB (signal recognition particle database). *Nucleic Acids Research*.

[40] Bharadwaj A. S., Appukuttan B., Wilmarth P. A. (2013). Role of the retinal vascular endothelial cell in ocular disease. *Progress in Retinal and Eye Research*.

[41] Liu X., Robinson M. L., Schreiber A. M. (2009). Regulation of neonatal development of retinal ganglion cell dendrites by neurotrophin-3 overexpression. *The Journal of Comparative Neurology*.

[42] Yoshida M., Feng L., Grimbert F. (2011). Overexpression of neurotrophin-3 stimulates a second wave of dopaminergic amacrine cell genesis after birth in the mouse retina. *Journal of Neuroscience*.

[43] McAllister A. K., Katz L. C., Lo D. C. (1999). Neurotrophins and synaptic plasticity. *Annual Review of Neuroscience*.

[44] Seki M., Fukuchi T., Tanaka T., Nawa H., Takei N., Abe H. (2004). Quantitative analyses of mRNA and protein levels of neurotrophin-3 in the rat retina during postnatal development and aging. *Japanese Journal of Ophthalmology*.

[45] Bates B., Hirt L., Thomas S. S. (2002). Neurotrophin-3 promotes cell death induced in cerebral ischemia, oxygen-glucose deprivation, and oxidative stress: possible involvement of oxygen free radicals. *Neurobiology of Disease*.

[46] Zhang J., Shi Q., Yang P. (2012). Neuroprotection of neurotrophin-3 against focal cerebral ischemia/reperfusion injury is regulated by hypoxia-responsive element in rats. *Neuroscience*.

[47] Ponto K. A., Scharrer I., Binder H. (2019). Hypertension and multiple cardiovascular risk factors increase the risk for retinal vein occlusions. *Journal of Hypertension*.

